# Optic Nerve Ultrasound Evaluation in Animals and Normal Subjects

**DOI:** 10.3389/fmed.2021.797018

**Published:** 2022-01-05

**Authors:** Livio Vitiello, Maddalena De Bernardo, Luigi Capasso, Palmiro Cornetta, Nicola Rosa

**Affiliations:** ^1^Eye Unit, Department of Medicine, Surgery and Dentistry, “Scuola Medica Salernitana”, University of Salerno, Baronissi, Italy; ^2^Corneal Transplant Unit, Azienda Sanitaria Locale (ASL) Napoli 1, Naples, Italy; ^3^Eye Unit, “Maria SS Addolorata” Hospital, Azienda Sanitaria Locale (ASL) Salerno, Eboli, Italy

**Keywords:** intracranial hypertension, optic nerve, optic nerve sheath diameter, ONSD, ultrasonography

## Abstract

In recent years, ultrasonographic measurement of the optic nerve sheath diameter (ONSD) has been widely used to identify the presence of increased intracranial pressure (ICP). Intracranial hypertension is a life-threatening condition that can be caused by various neurological and non-neurological disorders, and it is associated to poor clinical results. Ultrasonography could be used to qualitatively and efficiently detect ICP increases, but to reach this purpose, clear cut-off values are mandatory. The aim of this review is to provide a wide overview of the most important scientific publications on optic nerve ultrasound normal values assessment published in the last 30 years. A total of 42 articles selected from PubMed medical database was included in this review. Our analysis showed that ocular ultrasonography is considered to be a valuable diagnostic tool, especially when intracranial hypertension is suspected, but unfortunately this research provided conflicting results that could be due to the different ultrasound protocols. This is mainly caused by the use of B scan alone, which presents several limitations. The use of B-scan coupled with the standardized A-scan approach could give more accurate, and reliable ultrasound evaluation, assuring higher data objectivity.

## Introduction

Intracranial hypertension is a critical life-threatening condition caused by a variety of neurological and non-neurological diseases and it is associated with poor clinical outcomes and high mortality rates (1). One of the most representative sign of elevated intracranial pressure (ICP) is the presence of papilledema, characterized by disc elevation, blurred disc margins, venous congestion, hemorrhages, soft/hard exudates, and choroidal/retinal folds (1). The optic nerve is surrounded by the meninges and the space between the optic nerve sheaths and the optic nerve is connected to the subarachnoid space (2). In case of increased ICP, the subarachnoid fluid is pushed into the subarachnoid space surrounding the optic nerve, causing optic nerve sheath expansion (3).

In recent years, ultrasonographic measurement of the optic nerve sheath diameter (ONSD) has been widely used to identify the presence of increased ICP (4). Furthermore, ultrasound is a rapid, cheap, real-time, safe, and non-invasive diagnostic tool that could be really useful when the patients are critically ill, allowing a bedside appraisal. However, to perform a reliable and well-executed ocular ultrasonography, especially in the optic nerve evaluation, a well-trained and skilled operator is required, and a series of precautions in the exam execution and in the utilized probe and technique should be considered (5–7). Ultrasonography could be used to qualitatively and efficiently detect ICP increases, but to reach this purpose, clear cut-off values are mandatory, and some papers report conflicting results on the cut-off values and ultrasound protocols to identify intracranial hypertension.

Considering all the aforesaid reasons, the purpose of this review is to provide a wide overview of the most important scientific publications on optic nerve ultrasound normal values assessment published in the last 30 years, focusing on studies carried out in animal models and healthy volunteers, and to discuss the limitations of the most widely used B-scan technique.

## Materials and Methods

We searched within the PubMed medical database. A preliminary general Web search using Google was also performed in order to get a larger vision and understanding of the issue. We entered search strings including terms related to ocular ultrasonography, ICP, animals, and healthy subjects. Text words were chosen based on the existing literature and/or were obtained from related bibliographies as well. Bibliographies from the initial searches were manually searched for additional inclusions too.

Only English full articles and case reports or case series concerning optic nerve ultrasound evaluation in animals and healthy people were included in this study. The earliest publication date was set at January 1990, while the search ended in August 2021.

## Results

Our initial search yielded 137 results, of which 3 were excluded due to no English language. Subsequently, another 92 articles were excluded through successive reviews because they are not directly related to the discussed topic or because they were comments or observations to other articles. At last, 42 articles were included and divided into 2 subgroups: 12 articles concerned studies on animals, while 30 articles regarded studies on healthy subjects.

### Animals

Some studies were carried out in different animal models to evaluate the reliability of ocular ultrasound in the diagnosis of intracranial hypertension. Of the twelve articles on animals selected for this review, four were performed on pigs, four on dogs, one on cats, two on horses, and one on rabbits.

The aim, the utilized ultrasound technique and the main findings of these studies are summarized in the Table 1.

**Table 1:** Summary of published articles on ONSD ultrasound evaluation in animal models.

**Study**	**Aim**	**Animals**	**Ultrasound technique**	**Main findings**
**PIGS**				
Hamilton et al. (8)	To investigate if changes in the ONSD could consistently correlate with manipulated ICP	5 adult Yorkshire pigs with a fiber-optic intracranial pressure transducer placed into the brain parenchyma	ONSD ultrasound measurements acquired with B-scan technique on closed eyelids by two ultrasound operators, 3–5 mm posterior to the globe	Use of non-invasive ONSD ultrasound measurements could be considered feasible in a porcine model, confirming acute changes in intracranial pressure over 1 h
Nusbaum et al. (9)	To evaluate the utility of a porcine animal model of increasing cephalic venous pressure to mimic acute changes in ICP and ONSD from cephalic venous fluid shifts	10 healthy pigs and 20 pigs with elevated superior vena cava pressure (SVCP)	ONSD ultrasound measurements acquired with B-scan technique on closed eyelids, 2 mm posterior to the globe	Increases in SVCP result in ICP changes that are well correlated with ONSD alterations and that are consistent with observed ONSD changes
Mija et al. (10)	To measure the changes of the optic nerve, ONSD, and perineural space separately with increasing ICP in a porcine model	8 pigs with an external ventricular drain catheter	ONSD ultrasound measurements acquired with B-scan technique on closed eyelids, 3 mm behind the papilla	The optic nerve diameter correlates linearly with ICP and could be reliably measured using transbulbar ultrasonography. The ONSD increase could be mainly attributed to an increase of the optic nerve, while the diameter of the perineural space does not correlate with ICP
Jeng et al. (11)	To verify the correlation in an experimental porcine model of controlled ICP elevation by means of balloon inflation, along with interventions to reduce ICP, and observe ONSD	30 piglet hybrids of the Landrace, Duroc, and Pietrain breeds, with intraventricular catheter	ONSD ultrasound measurements acquired with B-scan technique on closed eyelids, 3 mm behind the optic disc	Ultrasound ONSD showed a linear correlation with ICP, although a short delay in returning to baseline levels may be observed in the case of sudden intracranial hypertension relief
**DOGS**				
Lee et al. (12)	To appraise the feasibility of ultrasonographic ONSD measurements in normal dogs and to assess the effect of breed, sex, body weight, and age on ONSD morphology	15 clinically healthy dogs (7 Yorkshire terrier and 8 Maltese)	ONSD ultrasound measurements acquired with B-scan technique on closed eyelids, 5 mm behind the optic disc	No statistically significant differences between ONSD and sex, body weight, and age was found, but there was significant difference within breeds
Ilie et al. (13)	To assess the association between ultrasonographically measured ONSD with B-scan and acute ICP increases	6 young healthy dogs with an epidural intracranial pressure monitoring system	ONSD ultrasound measurements acquired with B-scan technique on closed eyelids, 1–5 mm posterior to the globe	ICP is positively and non-linearly correlated with increasing maximum ONSD, suggesting that ultrasound maximum ONSD measurement could provide a non-invasive monitoring tool for ICP evaluation in dogs
Smith et al. (14)	To develop a reference range for ultrasonographically measured ONSD in dogs, also considering age, sex, weight, and body condition score	78 healthy adult dogs	ONSD ultrasound measurements acquired with B-scan technique on closed eyelids, 3 mm posterior to the globe	Age and body condition score both affect the ONSD, but body weight has the largest effect, with ONSD linearly increasing with increasing weight. An equation for calculating the prediction interval based on the combination of weight, age, and body condition score was developed
Armenise et al. (15)	To describe the technique and findings of a novel veterinary focused assessment with sonography for trauma-airway, breathing, circulation, disability, and exposure protocol in dogs suffering from trauma	64 dogs suffering from trauma	ONSD ultrasound measurements acquired with B-scan technique on closed eyelids, 3 mm posterior to the globe	Concerning ONSD, it is greater in dogs with a lower modified Glasgow Coma Scale score, and it decreased and coma score increased following mannitol infusion, which suggests that ONSD could be used serially to monitor response to therapy
**CATS**				
Evangelisti et al. (16)	To test the repeatability of ultrasonographic ONSD examination in the cat, the association between the ONSD and age, sex, and body weight in healthy cats, and the difference in the ONSD between healthy cats and those suffering from presumed intracranial hypertension	50 healthy cats and 7 cats with suspected intracranial hypertension	B-mode via the transpalpebral approach, with ONSD measured 3 mm posterior to the globe	This ultrasound technique is reproducible, non-invasive, and feasible to evaluate ONSD in healthy cats, and it could be considered very useful in diagnosing raised ICP in a feline model
**HORSES**				
Cooley et al. (17)	To describe the feasibility, repeatability, and reliability of ONSD ultrasound evaluation in a sample of clinically healthy horses, also investigating if ONSD has strong positive correlations with age and with body weight	48 horses (36 mares or fillies, 10 stallions or colts, and 2 geldings)	ONSD ultrasound measurements acquired with B-scan technique on closed eyelids, 3 mm posterior to the globe	Transpalpebral ultrasonographic ONSD measurement has acceptable intraobserver repeatability and interobserver agreement, while no correlation between ONSD and age or body weight in horses was found
Bramski et al. (18)	To determine whether there is an association between direct, invasive ICP measurement and indirect, non-invasive ultrasound ONSD measurement	8 clinically healthy adult horses with a placed ICP transducer	ONSD ultrasound measurements acquired with B-scan technique on closed eyelids	Although a weak to moderate positive association between direct ICP and ONSD measurements in adult horses under some conditions was found, the association was not consistent and was not present in standing horses
**RABBITS**				
Kasapas et al. (19)	To study the possible relation of the ultrasound neuromonitoring indices to the invasive ICP measurements in rabbits with induced epidural hematoma	20 adult New Zealand white rabbits	ONSD ultrasound measurements acquired with B-scan technique on closed eyelids, 2–3 mm posterior to the papilla. ONSD was measured as a “dark stripe” behind the globe	ONSD measurements were significantly related to invasive ICP increments in this experimental model of epidural hematoma in rabbits

### Healthy Volunteers

Considering the studies carried out on healthy subjects, eleven articles tried to establish the normal ONSD reference values, also focusing on possible effects of ethnicity, age, and sex, four articles concerned ONSD ultrasound accuracy and reliability, four articles focused on the effects of different body positions on ONSD, five articles discussed the effects of oxygen variations on ONSD, four articles evaluated the effects of cervical collar application on ONSD, while two articles assessed ONSD modifications due to physical activity.

#### ONSD Normal Range Values

Several papers on healthy participants tried to establish ultrasound ONSD normal reference values. Some of these studies were carried out on a limited number of patients (20–22), while others considered much more significant study samples (23–30).

Garcia et al. (20) assessed 23 healthy adults with coronal C-scan ultrasound, performing the exam with open eyelids and using topical anesthesia, showing a mean ONSD of 4.8 mm for the analyzed sample, while a mean ONSD of 4.9 mm in males and a mean ONSD of 4.5 mm in females were found.

Bäuerle et al. (21) appraised 40 healthy adults (18–77 years) with the same ultrasound protocol, proposing a value of 5.8 mm as threshold, with a specificity of 80%.

In a prospective observational cohort study on 50 Australian healthy pregnant women with uncomplicated singleton pregnancies, Kane et al. (22) performed a single prenatal ultrasonographic examination on all participants, with a postnatal examination performed on a subgroup with uncomplicated deliveries. The authors detected a mean ONSD of 4.34 mm, with no clear relationship with gestation or mean arterial pressure.

Ballantyne et al. (23) evaluated 102 healthy children with B-scan ultrasonography and closed eyelids, suggesting that an ONSD of >4 mm in infants <1 year, and 4.5 mm or greater in older children, should be considered as abnormal.

Maude et al. (24) carried out a prospective observational study on 136 Bangladeshi healthy subjects, finding out that ultrasonographic ONSD has a narrow bimodal distribution independent of gender, age, and head circumference, and an ONSD value greater of 4.75 mm should be considered abnormal in this population.

Chen et al. (25) tried to determine the ONSD reference values in a cohort of 519 healthy Chinese adults using B-scan ultrasound performed on closed eyelids. They found the median and the ONSD 95% percentile to be 5.1 and 5.9 mm, respectively. Moreover, they showed that ONSD was correlated with optic nerve diameter, while it was independent of gender, age, height, and weight.

Contrariwise, in another study carried out in Chinese population with the same ultrasound protocol, Wang et al. (26) appraised 230 healthy individuals, finding out the mean ONSD to be 3.46 mm, with 95% of participants in the range 3.42–3.49 mm, and to be correlated with sex and BMI. Furthermore, this study showed that the upper ONSD limit was lower than that one observed among Caucasian and African samples, also suggesting a potential ethnic/racial differences.

Concerning these possible ethnic differences, Kim et al. (27) studied ONSD ultrasound measurements in 585 Korean healthy volunteers, stating that obvious differences between Korea and other countries were not found, with a mean ONSD in their study sample of 4.11 mm.

Goeres et al. (28) assessed ultrasound ONSD measurements in a cohort of 120 healthy adult volunteers, stating that a lack of relationship to age, height, and weight was found. However, they found a difference depending on gender, suggesting the possible need for separate reference ranges for men and women. In fact, in this study, mean ONSD for men was 3.78 mm, compared with 3.60 mm for women.

Avci et al. (29) aimed to determine the standard ONSD value in 195 healthy adults aged 65 or older with no previous diagnosis of raised ICP. They compared right and left ONSD values and ONSD differences, according to the gender and age of the patients. In this study, the authors found out that the ONSDs of both eyes did not vary with age and gender in the study group, with a mean ONSD of 4.16 mm.

Focusing on potential ONSD differences related to sex and age, Chandrapatham et al. (30) evaluated 122 healthy individuals with B-scan ultrasonography, stratifying them for sex, and dividing them into three different age groups. The authors found ONSD to increase with age and to be greater in males (range: 3.9–4.6 mm) than in females (range: 3.6–4.2 mm).

The reference range values established by these studies are summarized in the Table 2.

**Table 2:** Summary of the optic nerve sheath diameter ultrasound reference range values established in studies on healthy people.

**Study**	**N**°** healthy volunteers**	**ONSD reference range values (mm)**		
		**Mean ± SD**	**Min**	**Max**
Garcia et al. (20)	23	4.80 ± 0.60	3.90	5.90
Bäuerle et al. (21)	40	5.40 ± 0.60	4.30	7.60
Kane et al. (22)	50	4.34 ± 0.40	Not reported	Not reported
Ballantyne et al. (23)	102	3.08 ± 0.36	2.10	4.30
Maude et al. (24)	136	4.41 (no SD reported)	4.24	4.83
Chen et al. (25)	519	5.10 ± 0.50	3.50	6.40
Wang et al. (26)	230	3.46 ± 0.28	2.65	4.30
Kim et al. (27)	585	4.11 ± 0.35	3.30	5.20
Goeres et al. (28)	120	3.68 ± 0.39	2.80	4.50
Avci et al. (29)	195	4.16 ± 0.69	2.20	6.20
Chandrapatham et al. (30)	122	4.20 (no SD reported)	3.60	4.90

*ONSD, optic nerve sheath diameter; SD, standard deviation*.

#### ONSD Accuracy and Reliability

In addition, different papers tried to establish the accuracy and the reliability of ONSD ultrasound appraisal in healthy people.

Betcher et al. (31) attempted to evaluate the ability of 23 military trainees to ultrasonographically measure the ONSD in healthy volunteers after attending a very brief training session, compared to four expert emergency physicians, concluding that trainees were able to perform ONSD with an accuracy similar to ultrasound experts.

Amini et al. (32) compared the traditional ultrasound visual axis technique to coronal axis technique for evaluating the ONSD in 42 healthy volunteers, concluding that the two ultrasound approaches were similar, with the coronal one slightly faster to perform and not technically challenging.

These results are in contrast with that ones by Blehar et al. on 27 healthy adults (33), who found that visual axis measurements do not reliably correlate with coronal axis measurements, with a statistically significant ONSD increase as the nerve coursed posteriorly when measured in the visual axis.

Cimilli Ozturk et al. (34) tried to define the operator variations in the ONSD ultrasound measurement performed on 60 healthy adults, utilizing the axial and the longitudinal approaches. They concluded that, although the levels of compatibilities for most of the measurements were found at acceptable levels statistically, ONSD sonographic measurement could not be considered a highly reliable method both in longitudinal and transverse planes due to the difficulties in the demonstration of the nerve sheath borders and small deflections in the gaze of eye direction.

#### ONSD and Body Position

In other three studies, the effect of different positions on the ONSD values were investigated.

Maissan et al. (35) carried out a study in which ONSD was measured by ultrasound in five healthy volunteers during helicopter liftoff and acceleration in the supine position or with a raised headrest. The authors found out that ONSD increased during helicopter acceleration from baseline, while after headrest elevation (20°-25°) the ONSD did not raise during helicopter acceleration. For this reason, the authors stated that ONSD and ICP seem to increase during helicopter transportation and, by raising the headrest of the gurney before liftoff, these effects could be prevented.

Romagunolo et al. (36) evaluated ONSD potential changes between the supine, Trendelenburg's, and reverse Trendelenburg's positions in 10 healthy people, discovering no statistically significant differences.

Inversely, Özdogan et al. (37) assessed the effect of spinal immobilization at 20°, compared to that one at 0°, on the ICP via the ONSD ultrasonographic measurement of 140 healthy adults, finding out that both these procedures increased ONSD.

Analogously, Pardon et al. (38) studied the ONSD differences over 12 h in seated and 6° head-down tilt postures in 30 healthy individuals, reporting no significant difference in sitting position, while ONSD increased during the head-down tilt posture.

#### ONSD and Oxygen Variations

Dinsmore et al. (39) examined the dynamic ONSD changes in response to mild fluctuations in cerebral blood volume induced by changes in end-tidal carbon dioxide on 11 healthy volunteers. A single investigator repeatedly measured ONSD for 10 min at each level of carbon dioxide: normocapnia (baseline), hypercapnia (6.5 kPa), normocapnia (baseline 1), hypocapnia (3.9 kPa), and on return to normocapnia (baseline 2). There was a significant ONSD increase with hypercapnia, while on return to normocapnia ONSD rapidly reverted back to baseline values, confirming dynamic ONSD changes with corresponding changes in carbon dioxide.

Some authors also studied the correlation between ONSD variations and acute mountain sickness (AMS) in healthy patients.

Keyes et al. (40) performed ultrasound ONSD measurements on 57 healthy subjects at 1,400 m and 18 h after rapid ascent to 4,300 m, both before and after oxygen treatment. They found that mean ONSD increased in subjects with AMS at high altitude, while individual variation was high, and most ONSD values were below the clinical threshold for raised ICP.

On the other hand, Di Pasquale et al. (41) manipulated exercise duration, barometric pressure, and ambient oxygen to assess 36 healthy volunteers before, during and after 8 h exposures in normobaric normoxia (300 m elevation equivalent), normobaric hypoxia (4,400 m equivalent), and hypobaric hypoxia (4,400 m equivalent). They assessed ultrasound ONSD measurements, documenting a small but significant increase in AMS patients, suggesting mildly elevated ICP, as well as further increased ONSD with longer exercise duration.

Kanaan et al. (42) evaluated 86 healthy adults enrolled at 1,240 m, drove to 3,545 m and then hiked to and slept at 3,810 m, performing ultrasound ONSD measurements before, the evening of, and the morning after ascent. They concluded that the mean ONSD increased on ascent to high altitude compared to baseline values, but not to a statistically significant degree, while the magnitude of the ONSD difference was positively associated with AMS diagnosis.

Strapazzon et al. (43) investigated changes in oxidative stress biomarkers and reactive oxygen species (ROS) during exposure to hypobaric hypoxia in 16 lowlanders, trying to correlate ROS related cellular damage and ultrasound ONSD as an indirect ICP measurement. Baseline measurement of clinical signs and symptoms, biological samples and ultrasonography were appraised at 262 m and after passive ascent to 3,830 m. Although ONSD was found to concurrently increase, regression analysis did not infer a causal relationship between oxidative stress biomarkers and ONSD changes.

#### ONSD and Cervical Collar Application

Maissan et al. (44) assessed the effect of application of a rigid cervical collar in 45 healthy volunteers by measuring their ONSD with ocular ultrasonography. Application of a collar resulted in a significant ONSD increase in both eyes, suggesting that ICP could raise after this application. This could be primarily related to the restrictive ability of the cervical collar around the neck vasculature. These same findings were confirmed by other authors (45–47), suggesting that clinicians should take proactive steps to assess the actual need of cervical collar case by case basis.

#### ONSD and Physical Activity

At last, two papers evaluated the correlation between ONSD and physical activity.

Sadrameli et al. (48) analyzed ONSD measurements of 24 female college soccer players during the initial visit in the pre-season period and at the 3-month follow-up. The authors found an ONSD increase from 4.14 to 5.02 mm, with an average ONSD measured during the post-season follow-up showing a 21.3% increase compared to the baseline.

Conversely, Lefferts et al. (49) measured ultrasound ONSD in 20 healthy participants at rest (baseline), following a time-control condition, and following acute high-intensity resistance exercise, showing no significant changes in ONSD.

## Discussion

In recent years, the use of ocular ultrasonography is spreading more and more in different medical fields, especially due to its safety and ready availability, making it easier for physicians to identify several pathological conditions (50). Specifically, this ultrasound technique is a non-invasive, non-irradiating, and cheap diagnostic tool that may be used to detect, indirectly, the presence of raised ICP. In fact, as shown in the present review, there are several scientific papers published in the literature in the last 30 years which describe the use of this ultrasound diagnostic method, also in animal models and in healthy people. However, except for one article (20), in all the other papers discussed in the present review, the authors utilized the B-mode ultrasound technique to evaluate ONSD as an indirect parameter to detect increased ICP.

Although nowadays B-scan ultrasonography could be considered highly sensitive to detect intracranial hypertension in some hospital settings, and it is often the only ready available diagnostic tool for such appraisals, it is crucial to point out some important pitfalls of this type of ocular ultrasonography, related both to how the examination is performed and to intrinsic limitations to the technique itself (51, 52). First of all, as highlighted by several papers discussed in this review, many authors tried to identify ONSD normal reference values, essential to diagnose ICP elevation. Nevertheless, these reference values were very contrasting and dissimilar in the various papers, as shown in the Table 2, with a real difficulty in determining them in an unambiguous way. This difficulty could be related to the so-called “blooming effect” which affects the B-scan ocular ultrasound (53, 54). This effect occurs when the equipment gain-setting is not standardized, in particular when performing repeated measurements over time. In fact, it is due to the absence of a standard gain and sensitivity setting: when a lesion is measured utilizing different gains, this will appear larger decreasing the gain, and smaller increasing it (55). Thus, considering the “blooming effect” and related less precise calipers location during ONSD evaluations, to examine very small structures, such as optic nerve, with B-scan ultrasound may not be objective and effective, providing potential bias and unreliable data (56).

In the early 70' Ossoinig introduced the Standardized A-scan technique (57), an ultrasound method equipped with an a 8 MHz non-focused probe, with a special S-shaped amplification, which is free of “blooming effect” and therefore permits more accurate measurements. Moreover, A scan shows easily discerned high-reflective spikes at the interface between arachnoid and subarachnoid fluid where the markers to measure such a structure can be easily placed, becoming even more important in case of follow up measurements (58–60). Furthermore, it is also important to remark how ocular ultrasonography should be performed to get more trustworthy ONSD measurements. Except for the study by Garcia et al. (20), in all the papers discussed in this review the echographic probe was positioned on the closed eyelids, making impossible to visualize the ocular globe and the patient's gaze direction. This could lead to errors during the ultrasound examination, providing less objective data (61). For this reason, the B- or A-scan probe should be usually used with open eyelids, utilizing methylcellulose and anesthetic drops, thus avoiding mistakes in the eye position visualization and making the probe orientation much more reliable (62).

In conclusion, ocular ultrasonography is certainly a powerful diagnostic device available to the physicians, especially in conditions where a potential intracranial hypertension is suspected. However, for a more complete, precise, and reliable ultrasound evaluation, B-scan ultrasound could be useful for a screening purpose, but it should always be associated with the standardized A-scan technique, to ensure greater data objectivity and accuracy provided by the examination.

## Author Contributions

LV, MD, and LC analyzed the literature and wrote the original draft. PC and NR conceived the article and reviewed the manuscript. All authors read and approved the final manuscript.

## Funding

The research was funded with the FARB grant from the University of Salerno.

## Conflict of Interest

The authors declare that the research was conducted in the absence of any commercial or financial relationships that could be construed as a potential conflict of interest.

## Publisher's Note

All claims expressed in this article are solely those of the authors and do not necessarily represent those of their affiliated organizations, or those of the publisher, the editors and the reviewers. Any product that may be evaluated in this article, or claim that may be made by its manufacturer, is not guaranteed or endorsed by the publisher.
